# Green Paradox in Emerging Tourism Supply Chains: Achieving Green Consumption Behavior through Strategic Green Marketing Orientation, Brand Social Responsibility, and Green Image

**DOI:** 10.3390/ijerph18189626

**Published:** 2021-09-13

**Authors:** Muhammad Ishfaq Khan, Shahbaz Khalid, Umer Zaman, Ana Ercília José, Paulo Ferreira

**Affiliations:** 1 Department of Management Sciences, Capital University of Science and Technology, Islamabad 44000, Pakistan; ishfaq@cust.edu.pk (M.I.K.); mshahbazkhalid765@gmail.com (S.K.); 2 Endicott College of International Studies, Woosong University, Daejeon 34606, Korea; 3 Department of Economic Sciences and Organizations, Polytechnic Institute of Portalegre, 7300-555 Portalegre, Portugal; anajose@ipportalegre.pt (A.E.J.); pferreira@ipportalegre.pt (P.F.); 4 VALORIZA—Research Center for Endogenous Resource Valorization, 7300-555 Portalegre, Portugal; 5 CEFAGE-UE, IIFA, University of Évora, 7000-809 Évora, Portugal

**Keywords:** green supply chain management, strategic green marketing orientation, green image, brand social responsibility, green consumption intention, signaling theory

## Abstract

Globally, green consumption behavior has radically changed green product lifecycles as well as green product branding to eliminate the environmental impact of global tourism. The purpose of the current study is to examine green consumption intention in the hospitality and tourism industry as an outcome of green supply chain management and strategic green marketing orientation. It also aims to investigate the green brand image and green social responsibility in a mediated-moderation mechanism to induce green consumption. Based on the deductive approach, and cross-sectional quantitative data of 317 hotel visitors/guests in the northern tourism hotspots in Pakistan, the hypothesized relationships were tested through the structural equation modeling technique with partial least squares. The findings empirically establish that green supply chain management and strategic green marketing orientation have positive and significant effects on green consumption intention. Further, environmental concern (i.e., green image) partially mediates the relationship between strategic green marketing orientation, green supply chain management, and green consumption behavior. The results also revealed that brand social responsibility does not moderate green image and green consumption behavior. These stimulating new findings guided by the signaling theory, provide strategic insights that help to upgrade the tourism supply chains and enabling them to become green.

## 1. Introduction

The global tourism supply chains have been blamed for causing approximately eight percent of the worldwide carbon emissions. The hotel supply chain was affected by the outbreak of COVID-19. Due to emerging competition in the tourism industry, hotels are required to provide better services than their competitors and ensure customer satisfaction [1]. The World Travel and Tourism Council issued a warning that due to COVID-19, 50 million employees may lose their jobs around the world in the Tourism industry [2]. The COVID-19-enforced complete lockdown reduced the raw material supply, imposed traveling restrictions, banned hotels opening for the public, etc. [3]. The supply chain in hotels is an organizational network that functions through the flow of information such as room reservations, payment transactions, physical items (food and drinks). The reverse transaction direct supply chains from customers to hotels (recycling and reuse) are part of hotel supply chains [4]. The traditional practices of the hotel and tourism industry continuously pose a significant threat to the environment, e.g., monoxide emissions, disposable package materials, scrapped toxic material, traffic congestion, and other forms of industrial pollution [5]. The hospitality industry needs to develop environmental management strategies to cope with the environmental requirements and their impact on supply chain operations [6]. This is also because the interest of the consumers with regards to the protection of the environment has been revitalized [7,8,9,10,11,12,13,14,15,16]. Ghosh (2010) also suggested that increased public attention and awareness have caused manufacturers to be environmentally responsible [17]. Further, increased governmental pressures, competitive pressures, cost issues, and increased social responsibility have driven hospitality businesses to incorporate green environment concerns into their core business processes [8,12,13,15,18]. Traceability and monitoring of these processes are becoming more critical to assure the finished product value by using blockchain technology that allows designers, sellers, and final customers to track the shipments [19]. The researchers acknowledge that green marketing research is a critical issue related to the environment and needs to be addressed as a priority [16,20].

There have been many supply chain management challenges revealed in multiple studies, such as logistics and transportation including reducing travel distance, time, cost, and environmental impact. In this situation, routing software may deal with the problem but it may also face some constraints that include traffic congestion, vehicle speed limits, transportation regulations, and restricted zones. Further, these constraints affect time-dependent vehicle-routing problems (VRP), VRP with time windows, dynamic VRP, and green VRP [21,22]. To reduce the environmental and social impact, the proper use of traffic congestion information can significantly improve public confidence in reduced traffic and confidence in the road network [23]. Research studies focused on the need for identification and evaluation of green innovation criteria for adopting sustainable supply chain management (SSCM) activities, and other environmental issues like global warming, the scarcity of natural resources, and climate change [24]. The identified gap filled by research that investigated and analyzed the green innovation criteria for SSCM, and the selection of suppliers who can deploy green SSCM by developing an integrated multi-criteria decision-making (MCDM) model using fuzzy analytical hierarchy process (FAHP) and the fuzzy technique for order of preference by similarity to ideal solution (FTOPSIS) [25]. Supply chains (SCs) are increasingly perceived to be at the heart of societal challenges: these can be addressed using critically engaged research in SCM to build, elaborate, and test theory [26]. The study revealed that multicriteria decision-making (MCDM) methods are used for analyzing the barriers, challenges, drivers, enablers, performance and practices of SSCM [23]. In addition to these aspects of the supply chain, the researchers also conducted many studies on environmental themes and intentional consumption behavior, e.g., natural airborne TiO2 [27]; ecology [28]; corporate social responsibility [29]; attitude [30]; sustainable consumption [31]; sustainable production characteristics [32]; premium price [33]; psychographic factors [34]; green brands [35]; knowledge [36]; energy efficient home appliances [37]; consumers attitude [38]; psychological climate [39]; eco-label credibility [40]; culture [41]; eco-friendly food behavior [42]; green advertising [43]; emotional factors [44]; lifestyle [45]; greenness exposure [46]. However, rare research studies are found on green supply chain management, strategic green market orientation, corporate social responsibility, green image, and green consumption intention. Similarly, consumption intention and environmental research are conducted in different regions of the world, e.g., environmental attitude in Mexico [47]; willingness to pay in Japan [48]; premium price in Malaysia [49]; eco-labeled products in Turkey [50]; organic foods in Taiwan [51]; cultural values in Vietnam [52]; spirituality in India [53]; Islamic values in Iran [54]; consumer perceived readiness in Indonesia [55]; social media in the USA [56]; religious values and habits in Indonesia and Malaysia [57]; design of energy labels in Switzerland [58]; smart home objects in France [59]; green consumerism in Slovenia [60]; fast-moving consumer goods in Germany [61]. There are rare studies found on environmental perspectives in Pakistan, e.g., green entrepreneurship [62]; factors determining green behavior [63]. Ali and Ahmed (2012) argued that research on environmental perspectives in hospitality is scarce in the context of developing nations such as Pakistan [7]. The current study examines the linkage between green supply chain management, strategic green market orientation, corporate social responsibility and green image constructs to enhance the body of knowledge from an environmental perspective of green consumption intention in the hospitality and tourism industry of Pakistan.

## 2. Literature Review

The literature of previous studies is reviewed from a green consumption perspective. The constructs such as green supply chain management, strategic green marketing orientation, green brand image, and brand social responsibility are explained below:

### 2.1. Green Supply Chain Management (GSCM) and Green Consumption Intention

GSCM is defined in respect of environmental concerns [64]. Garcia et al. (2020) argued that green supply chain management (GSCM) involves a combination of various facets that include product development and design, procurement of raw materials and their selection criterion, manufacturing procedures, product transportation, and the disposal of products after their shelf life has expired [65]. GSCM can be termed as a hotel’s plan to acknowledge and adopt environmental planning across the supply chain process in order to significantly boost its environmental footprint [66]. Hsu and Hu (2009) have defined green supply chain management as the evaluation process of the suppliers that is focused on their environment [67]. The hotels are now aiming to gain a competitive advantage by integrating GSCM into their long-term strategic planning processes [68]. Furthermore, green supply chain management (GSCM) has been acknowledged (by Micheli et al., 2020) as a vital hotel management strategy to improve overall environmental sustainability that increases the hotel’s profits and accomplishes market share objectives successfully and effectively assimilates the associated environmental threats and impacts [69]. The studies revealed that partnership termination is concerned with the action that a supplier fails to meet the prescribed environmental regulations and procedures [66,67,70,71,72,73,74,75].

The hotel’s green activities lead to a significant improvement in environmental sustainability and performance [76,77]. Further, environmental partnership management between the supplier and purchaser is also important as it provides an opportunity for both entities to learn the environmental goals of the other [64,77,78,79,80,81,82]. It was also posited that a green supply chain can significantly enhance the hotel’s overall reputation and financial performance due to the reduction in costs, energy consumption, and management of waste material [65,76,83,84]. Han and Huo (2020) and Bu et al. (2020) observed that the integration of a green supply chain had a positive relationship with the sustainable performance of the firm [81,84]. Wong et al. (2012) posited that irresponsible behavior impacts the reputation and brand image of the hotels [83]. Hence, hotels are now actively investing in the development of green practices.

Green supply chain management (GSCM) can aid hotels in achieving sustainability by reducing pollution and production costs. This will lead to the development of a positive and strong brand image amongst the masses. The customers recognize and perceive the green initiatives of the hotels as a part of its corporate social responsibility activities [16]. GSCM constitutes an important part of the decision-making process in hotels and therefore, its link with strategic marketing planning cannot be neglected [85]. The integration of green supply chain management practices positively shape the perception of the consumers towards the hotel. Hence, in light of the above-mentioned findings, this study hypothesizes the following relationship:

**Hypothesis** **1** **(H1).** *Green supply chain management has a positive and significant relationship with green consumption intention.*

### 2.2. Green Supply Chain Management and Hotel’s Green Image

A hotel’s green image usually involves the combination of a hotel’s concern with regards to the image of its operational activities as being environmentally friendly. Moreover, a green hotel image is established when the customers of a hotel are aware of the hotel’s environmentally friendly actions and products [13]. Kumar et al. (2018) stated that the pursuance of green strategies could yield positive results, such as the development of a hotel’s positive reputation, profit maximization and enhancement in overall hotel image [86]. Testa & Iraldo (2010) stated that the main underlying reason behind the inculcation of green strategies is to improve green corporate image and to gain a competitive edge [87]. The green hotel image embodies perceptions of the stakeholders towards the environmental actions and practices of a hotel [17,87,88,89,90,91,92,93,94,95].

Many studies revealed that green supply chain management (GSCM) is a vital factor that plays a critical role towards the development of a hotel’s reputation [85]. The adoption of green supply chain practices, such as eco-friendly products, services, and eco-friendly transportation, significantly enhance its overall green image [12]. Environmentally friendly practices help in shaping positive consumer perceptions about the hotel and consumers start to perceive the hotel as being more responsible towards the natural environment [96,97]. Further, Aslam et al. (2019) also undertook a study to investigate the impact of GSCM practices on corporate image. It was observed that GSCM practices had a positive and significant relationship with corporate image [85]. Hence, in light of this evidence, the current study hypothesizes the following relationship:

**Hypothesis** **2** **(H2).** *Green supply chain management has a positive and significant relationship with green image.*

### 2.3. Strategic Green Marketing Orientation and Green Consumption Intention

Strategic green marketing orientation (SGMO) is a strategic initiative of the top management aimed at focusing on a hotel’s environmental strategy, external environmental factors, proactive environmental efforts and environmental sustainability [17,98,99,100,101,102]. The greening of techniques and processes refers to the steps taken by a hotel to improve its existing processes by eliminating wasteful activities and reducing the consumption of additional resources [103,104]. On the other hand, Crane (2011) argued that hotels that exhibit minimal to no interest in green marketing are prone to face difficult strategic choices. In a niche green marketing strategy, the hotel positions itself and its products as green alternatives as compared to those being offered by its competitors [105]. Collaborative greening involves an integrated approach that includes muted, passive and niche green marketing and is focused on innovation, newness, creativity, product orientation, and market orientation [16].

The hotel’s traditional marketing perspective of boosting sales and consumption adversely impacts overall environmental sustainability [106,107]. Zhu & Sarkis (2004) argued that hotels are required to implement green environmental practices in order to enhance their overall level of environmental performance [108]. According to Delmas and Montiel (2007), many hotels require their supply chain partners to comply with the pre-established environmental rules and regulations [96]. Strategic green marketing orientation is a comprehensive management mechanism that involves the process of identifying, analyzing, and catering to the needs of the consumers in a sustainable manner [16]. Green marketing orientation is a strategic mechanism that inculcates environmental concern into the core business processes and organizational practices [97]. According to Oyewole (2001), the green initiatives undertaken by hotels will send a signal to the consumers to actively engage themselves in the consumption of green hotel products [109]. Empirical studies revealed a significant relationship that exists between green marketing orientation and consumer purchase intentions [16]. Moreover, Maheshwari (2014) in his study on Indian consumers observed that the hotels’ green marketing efforts and initiatives will drive the consumers to consume green products and services [97]. Hence, in the light of these findings, the present study hypothesizes the following relationship:

**Hypothesis** **3** **(H3).** *Strategic green marketing orientation has a positive and significant relationship with green consumption intention.*

### 2.4. Strategic Green Marketing Orientation and Hotel’s Green Image

Many studies have examined whether the hotels that are engaged in proactive environmental marketing strategies tend to have a competitive edge over their competitors [16]. The hotels that adopt and implement sustainable marketing strategies are more innovative, consumer-oriented and socially responsible than those that do not adopt such strategies [110]. Maheshwari (2014) observed that by enhancing environmental efficiency, the hotels could achieve certain positive outcomes such as reduced costs, increased performance, increased loyalty, and improved corporate image [97]. Similarly, Leonidou et al. (2013) also posited that green marketing strategies could aid the hotels to bring about a significant enhancement in their overall reputation [111]. 

Menguc et al. (2010) argued that by adopting green marketing strategies, the organizations could send a positive signal to the consumers and hence they can present themselves as environmentally responsible [110]. Therefore, it can be ascertained that strategic green marketing orientation is a major precursor and antecedent of green image. Hence, the present study deduces the following relationship:

**Hypothesis** **4** **(H4).** *Strategic green marketing orientation has a positive and significant relationship with green image.*

### 2.5. Hotel’s Green Image and Green Consumption Intention

A hotel’s green image usually involves the combination of a hotel’s concern with regards to the image of its activities being perceived as environmentally friendly. Moreover, a green image is established when the customers are actively aware of the hotel’s environmentally friendly actions and the consumption intention of its products [13]. Green consumption intention involves the efforts and actions undertaken by the consumers to minimize the adverse effects of consumption on the surrounding environment [48,93,112,113,114,115,116,117,118,119,120,121,122]. There is still a dearth of research when it comes to examining the role of a hotel’s green marketing orientation practices in fostering individual green consumption behavior. Xu et al. (2020) stated that contemporary environmental problems like climate change, pollution, etc., have played a key part in fostering the development of environmental awareness among consumers [123]. These problems have induced the consumers to act in an environmentally responsible manner by engaging themselves in certain pro-environmental behaviors. According to Maniatis (2016), these attitudes in turn, play a significant role in stimulating the consumers to display green consumption behavior by involving themselves in the buying of green products [117]. Research by Xu et al. (2020) suggested that a hotel’s green marketing practices and green image were major drivers of green consumption intention amongst the consumers [123]. Green consumption intentions arise as a result of eco-friendly attitudes resulting from enhanced levels of environmental awareness [124]. Wu and Yang (2018) also noted a positive association between organizational strategy and green consumption intention [125]. Chuang and Huang (2018) in their study on Chinese consumers noted that increased environmental awareness amongst the consumers led them to the consumption of eco-friendly products [126]. Lee et al. (2010) further posited that green image significantly influences positive word-of-mouth, intention to repurchase and a willingness to pay a premium price for a green product [90]. According to Huang et al. (2014), the green positioning of the hotel enhances the green knowledge of the consumers, which leads to the development of green consumption intentions [127]. However, there is still a need to thoroughly examine the association and linkage between green image and green consumption intention [128,129,130]. Hence, it becomes imperative to undertake a study aimed at explaining the impact of a hotel’s strategic green marketing orientation and green supply chain management practices on green consumption intention through the mediating role of the hotel’s green image.

A hotel’s green image has received increased attention recently due to the increase in environmental issues that have directed hotels to adopt more environmentally sustainable practices [13]. As a result, the consumers tend to associate themselves with such hotels by depicting certain behavioral outcomes. According to Kumar et al. (2018), the hotels that actively engage and invest in making their practices more eco-friendly are perceived by the consumers as more responsible and environmentally-friendly [86]. These behavioral outcomes include trust, loyalty, and purchase behavior. Moreover, Han and Huo (2020) in their study examined the impact of green image on the purchase intentions of Chinese consumers [84]. The results suggested that corporate image played a positive role in shaping overall consumer intention. Hence, it can be ascertained that green image is a major precursor of consumer purchase intention. Therefore, it is hypothesized that:

**Hypothesis** **5** **(H5).** *Green image has a positive relationship with green consumption intention.*

### 2.6. Hotel’s Green Image as Mediator

In the current competitive world, where the consumers are more aware and conscious about their surrounding environment, the hotels are taking steps that are aimed at maintaining a more environmentally responsible corporate image. Many studies have also indicated that a green image aids in fostering positive consumer behavioral outcomes [13,84]. For instance, the impact of green practices on purchase intention is analyzed through the mediating role of green image [16]. The results revealed that green image plays a mediating role between green practices and customer purchase intentions. Moreover, Han and Huo (2020) in their study on the Chinese consumers also observed that green image is a major antecedent of green customer purchase behavior [73,84].Furthermore, Sarkis and Zhu (2018) in their study examined the impact of green supply chain management (GSCM) practices on green customer satisfaction through the mediating role of green image [13]. Therefore, the present study intends to examine the following mediation hypotheses:

**Hypothesis** **6** **(H6).** *Green image mediates the relationship between strategic green marketing orientation and green consumption intention.*

**Hypothesis** **7** **(H7).** *Green image mediates the relationship between green supply chain management and green consumption intention.*

### 2.7. HotelBrand’s Social Responsibility as a Moderator

Consumer confidence builds when a hotel fulfils its legal requirements, economic standards and policies [131]. Carroll (2016) argued that corporate social responsibility includes the voluntary activities of a hotel which reflect its involvement in community welfare work through providing sponsorships and donations [132]. Kotler and Lee (2005) explain the concept of CSR as a way of improving the society where we are living [133]. Jiang et al. (2015) performed an in-depth work on CSR in which they linked CSR with sustainable development [134,135]. It has been acknowledged by Sarkis and Zhu (2018) that, with the passage of time, CSR has affected consumers in a very positive way [13]. Now consumers are becoming more contented and satisfied with the hotels that engage in CSR activities that are practiced for the sake of community welfare [136]. Hence, it becomes imperative for the hotels to appropriate significant resources for effectively planning and executing CSR-related activities in order to strengthen their hotel image and at the same time drive the consumers to develop consumption intentions for the products and services.

According to Burke and Logsdon (1996), a hotel’s social responsibility is a value creation tool that encompasses the offering of products and services by integrating them with societal issues [137]. Little (2006) posits that CSR initiatives can lead to the generation of innovative work processes that aid in effectively responding to various social, economic, and environmental needs [138]. CSR-driven hotels are more likely to drive consumers to engage with the firm in a constructive manner [16]. According to Kilic and Ozdemir (2018), a hotel’s social responsibility is a major precursor that aids in fostering customer buying behavior. Similarly, the fulfillment of CSR-related activities leads to the development of positive consumer evaluations of the hotel [138]. Moreover, Attaran and Celik (2015) also observed that CSR has a positive and direct effect on the buying behavior of consumers [124]. Fatma and Rahman (2016) in their study on Pakistani consumers also emphasized the fact that social responsibility acts as a catalyst in fostering consumer buying behavior [139]. Hence, keeping in view the findings mentioned in the above literature, this study posits that brand social responsibility will strengthen the relationship between green image and green consumption intention. Therefore, the following hypothesis has been presented:

**Hypothesis** **8** **(H8).** *Brand social responsibility moderates the relationship between green image and green consumption intention such that it strengthens the association.*

### 2.8. Signaling Theory

**Figure 1:**
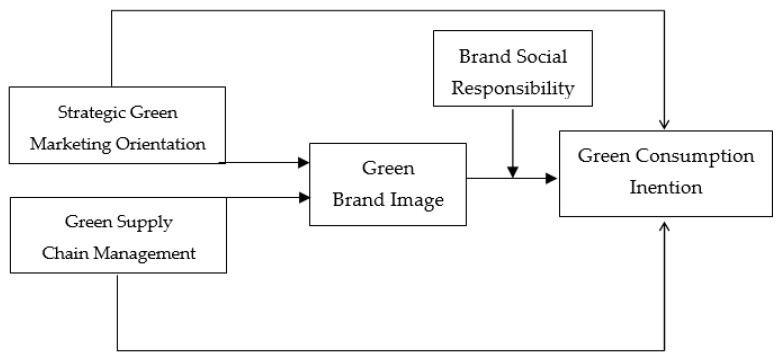
Theoretical framework.

The current study examines the aforementioned hypotheses (i.e., proposed relationships reflected as Figure 1) through the lens of the signaling theory. The signaling theory has been widely acknowledged and used in the green marketing literature [11]. The signaling theory presents a valuable framework that significantly aids in understanding the various antecedents and precursors that drive the green consumption behavior of consumers [140]. Therefore, in order to ensure the adequate availability of information, hotels attempt to convey information to the consumers with the aid of certain signals. The hotel is the sender of signals and the consumers are the receivers of the signals [11]. The behavioral outcomes of the receivers are a direct function of the nature of the signals. It means that positive signals tend to yield positive results and vice versa [12].

The researchers tend to agree over the fact that the environmental practices of the hotels are a major signal that is received by the consumers and upon the basis of these signals, the consumers tend to form a certain perception about the hotel. These perceptions, in turn, drive the consumers to depict certain behaviors, such as green purchase and consumption behavior [16]. Furthermore, Fatma and Rahman (2016) also observed that a hotel’s social responsibility also acts as a signal that sends crucial information to the relevant stakeholders of the firm, such as its consumers [139]. Therefore, in the context of the current study, the signaling theory can be applied in a manner that the green initiatives of hotels such as strategic green marketing orientation, green supply chain management and green image will send a positive signal to the consumers and upon the basis of those signals, the consumers will view that hotel as more environmentally responsible and hence, they will engage with that hotel by adopting green consumption intention towards its products.

## 3. Methodology

Based on the deductive approach, the cross-sectional research design was used and data were collected from hotel visitors in northern regions of Pakistan. The population of the current study comprised of visitors/tourists to hotels in the northern regions of Pakistan. The survey was conducted from 4 April 2021 to 15 June 2021. Using non-probability sampling, the Google form link was sent via emails sent to 1700 hotel visitors and northern region tourists, and 370 respondents responded to the survey. A total of 53 samples were deleted as they have replied “NO” to the criterion question, “Have you visited the northern region of Pakistan?”. Thus, 317 respondents’ data were used in the statistical analysis and found suitable as a sample size larger than 200 was considered safe for generalization [141]. The measurement scales for the present study were adopted from prior studies that have been conducted in similar contexts. The measurement scale is comprised of two sections. The first section included demographic information such as age, gender, qualification, income levels. The second section consisted of the statements that were used to measure the constructs. Strategic green marketing orientation (SGMO) was measured using 6 items adopted from scale developed by Papadas et al. [18]. Green supply chain management (GSCM) had 8 items and its scale was adopted from Çankaya and Sezen [142]. The green image was measured using 5 items adopted from Chen [143]. There were 4 items of green consumption intention and its scale was adopted from Sheng et al. [144]. Moreover, the scale of brand social responsibility consisted of 7 items adopted from existing well-established scales [132,133,134,135,136,137,138,139].

The current study used statistical software for the purpose of arranging, organizing, and analyzing the data that was obtained from the respondents. Linkages between the constructs proposed in the theoretical framework were examined with partial least square structured equation modeling (PLS-SEM) in SMART PLS 3.0 (SmartPLS GmbH, Boenningstedt, Germany).

## 4. Results Analysis

This section sheds light on the results that were obtained after a rigorous analysis using various quantitative and statistical procedures. Firstly, a demographic analysis was undertaken in order to ascertain the various demographic characteristics of the participants. These characteristics included gender, age, education, and income of the participants. This was followed by descriptive analysis. After this, a thorough examination of the measurement and structural model was carried out using a structured equation modeling (SEM) technique using SmartPLS software. The statistical tests included construct reliabilities, factor loading, correlation, regression, path analysis for mediation, and a moderation test. The results of the analysis have been summarized at the end of this section. On the basis of the results, some key conclusions and recommendations have been formulated and presented in the succeeding section.

### 4.1. Demographic Characteristics

The survey forms that were distributed to the participants comprised of two sections. The first section consisted of the information pertaining to the demographic characteristics of the participants. These traits included: gender, age, education, and income levels. The second section of the survey form included questions pertaining to the constructs of the study. The responses were gauged using a 5-point Likert scale. These responses were then utilized to ascertain the relationships between the constructs of the study.

Table 1 demonstrates the distribution of the respective genders of the tourists who participated in the study. It can be seen that a total of 196 male tourists participated in this study which comprised 61.8% of the entire sample. Moreover, it can also be observed that 121 female tourists also took part in this study which constituted around 38% of the entire study sample. The majority of the respondents were males, although significant and observable participation was witnessed from the females as well. Similarly, with age distribution it can be observed that 139 respondents fell within the age bracket of 20–30 years and constituted 43.8% of the total representative sample. Some 150 tourists were aged between 31–40 years and made up the majority of the study sample, i.e., 47%. Twenty respondents belonged to the age group of 41–50 years and constituted around 6% of the total sample. Moreover, only eight participants were aged between 51–60 years and they comprised 2.5% of the entire sample. The distribution of the education levels of the participants showed that 129 respondents possessed a high school education and they constituted 40.7% of the total representative sample. A further 118 participants had an undergraduate education and comprised 37.2% of the total sample size. Moreover, 70 respondents had a postgraduate degree and they constituted 22.1% of the entire sample. Similarly, the distribution of the respective income levels of the study’s tourist participants showed that 168 participants had an income of less than PKR 50,000 and they made up 53% of the representative sample. Ninety-four participants had an income of PKR 50,000 to 100,000 and they comprised 29.7% of the study sample. Thirty-four tourists had an income that ranged between PKR 100,000 and 200,000 and they constituted 10% of the study sample, whereas, only 21 participants had an income of more than PKR 200,000 and they comprised only 6.6% of the entire representative sample.

Table 2 demonstrates the descriptive statistics that include the mean or average values along with their standard deviations. The values for skewness and kurtosis can also be witnessed. It can be seen that all the mean values of the constructs were greater than 3.00, which means that the average responses against these constructs were inclined towards the 4.00 on the Likert scale (i.e., agree). The standard deviation is a measure of the degree or extent of dispersion around the mean values. It can be observed that all values of standard deviation fell within the acceptable threshold limit of −1 to +1. It means that the data is normally dispersed around the mean. Skewness and Kurtosis are the measurements of data normality. These measurements depict whether the data is normally distributed or not. It can be observed from Table 2 that all the values of skewness and kurtosis were well within the threshold limits of −1 to +1 and −3 to +3, respectively. Hence, it can be ascertained that the data were normally distributed and therefore fit for the undertaking of further statistical analysis.

### 4.2. Measurement Model

The first step in the evaluation of the measurement model included the assessment and evaluation of the outer PLS model. It consisted of the analysis of the main facets that constituted the main model. There are two main dimensions of the outer PLS Model. These dimensions are reliability and validity. The guidelines pertaining to the assessment of the measurement model include internal consistency reliability in between the items of the constructs through the aid of Cronbach’s alpha and composite reliability (CR), average variance extracted (AVE), convergent validity, and discriminant validity. The validities were measured using the Fornell−Larcker method [145,146].

Table 3 demonstrates the factor loadings of the items that were used to gauge the responses from the tourists. Measurement loadings are the standardized path weights connecting the factors to the indicator variables. Smart PLS standardized data loading from 0 to 1 indicator reliability is interpreted as the square of the measurement loading that needs to be ≥0.5 [147]. According to Santos (1999), the acceptable threshold value for Cronbach’s alpha is 0.70 [134]. It can be observed that all the internal consistency reliability values were greater than 0.70. Hence, it can be said that the measures adopted for this study are highly reliable.

Unlike Cronbach’s alpha, composite reliability (CR) does not account for equal loading of a particular construct. The range values of composite reliability lie between 0 and 1. The acceptable and satisfactory CR value should be greater than 0.60. A composite reliability score ranging between 0.60 and 0.70 is considered to be satisfactory, whereas a CR score ranging between 0.70 and 0.90 is regarded as highly acceptable and desirable. It can be observed from Table 3, given above, that all the CR scores against each of the constructs are greater than 0.70. Hence, it can be ascertained that the measurement model of this study is highly reliable for conducting further statistical analysis. After the assessment of internal consistency reliability and composite reliability, the next procedure is to assess the presence of convergent validity. Convergent validity is the extent to which the constructs of the study have a certain theoretical relation with each other.

Average variance extract (AVE) is the measure of the degree of convergence between the under-studies. The minimum acceptable and desirable value for AVE should be more than 0.50.The table shown above sheds light on the AVE values that have been obtained against each of the constructs. The range of the AVE values lies between 0.562 and 0.746. Therefore, it can be ascertained that convergent validity is present within the constructs.

Discriminant Validity can be termed as the extent to which one particular construct is unique and different from the other. The Fornell−Larcker method is the most widely acknowledged approach used to measure discriminant validity [19].

Table 4 demonstrates the discriminant validity scores obtained using the Fornell−Larcker approach. It can be observed that the square root of the AVE scores of a construct was greater than the construct highest correlation within another latent variable.

For instance, BSR had a score of 0.706 that was greater than the scores of the other constructs (GCI, GI, GSCM, and SGMO). GCI had a score of 0.737 that was greater than the score of other constructs (GI, GSCM, and SGMO). GSCM had a score of 0.807 that was greater than the score of SGMO. Therefore, it can be deduced that the discriminant validity is present between the constructs of the study.

### 4.3. Structural Model

**Figure 2:**
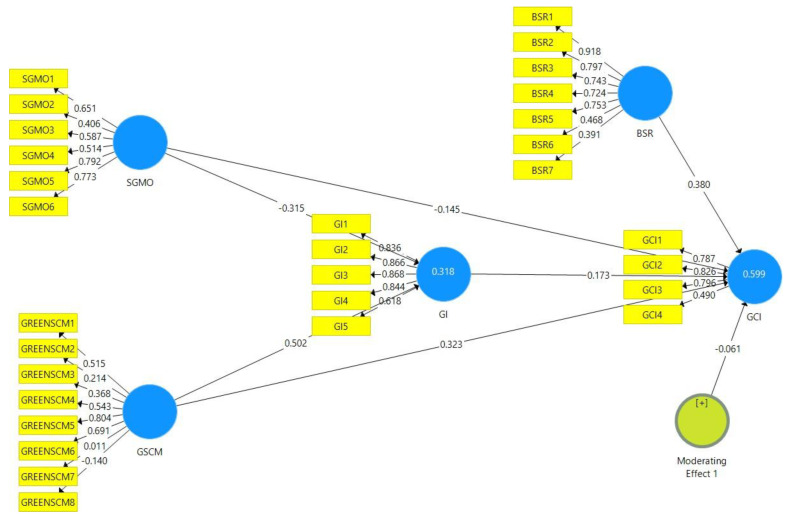
PLS-SEM algorithms ‘direct relationships.

After a thorough examination of the measurement model, the next phase involved the assessment of the structural model. This study undertook a detailed examination of the structural model shown in Figure 2.

The first phase involved the examination of the direct relationships between the constructs. A PLS-SEM bootstrapping technique was utilized to assess and examine the coefficient size and the *p*-values. The bootstrapping re-sampling procedure involved the use of 95% bias-corrected bootstrap intervals that included 317 subsamples. This was done in order to assess the impact of the two predictor variables (SGMO and GSCM) on the outcome variable (GCI). 

**Figure 3:**
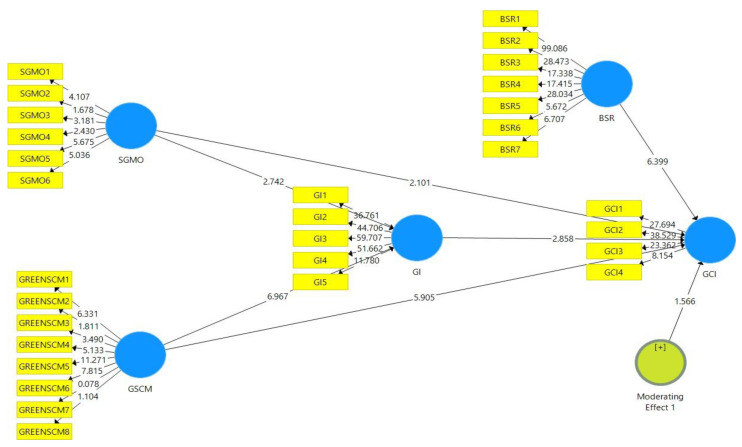
PLS-SEM bootstrapping direct relationships.

Moreover, the standard errors, path coefficients and t-statistics were also analyzed in order to ascertain the validity of the proposed hypotheses. Figure 2 is a depiction of the PLS-SEM algorithm (Figure 2) and the bootstrapping direct relationship model (Figure 3) that were used to determine the relationship between the constructs of the study.

Table 5 below depicts the results that were obtained after using a PLS-SEM algorithm and the bootstrapping direct relationship technique. The inner model analysis included original sample means, standard deviation, *t*-statistics and *p*-values. It can be observed that all values of the t-statistics surpassed the minimum acceptable limit of 1.96. Therefore, it can be ascertained that all the outer model loadings were significant. The results revealed that the first Hypothesis H1 has been accepted as indicated by (*t* value = 1.96 and *p*-value = 0.006). It means that there is a positive link between strategic green marketing orientation (SGMO) and green consumption intention (GCI). The signaling theory presented a valuable framework that significantly aids in understanding the various antecedents and precursors that drive the green consumption behavior of consumers [140]. The second Hypothesis H2 has also been accepted as shown by (*t* = 2.742; *p* = 0.006). It can be deduced that a positive association exists between SGMO and green image (GI). An identity built around corporate sustainability sends signals to the consumers’ that improves their beliefs in terms of benefits [145].

The Hypothesis H3 is also accepted as suggested by (*t* = 7.961; *p* = 0.000). It means that a positive relationship exists between GSCM and GCI. The fourth Hypothesis H4 that proposed a positive relationship between GSCM and GI has also been accepted as indicated by (*t* = 6.967; *p* = 0.000). The fifth Hypothesis H5 proposed a positive relationship between GI and GCI. This hypothesis has also been accepted as depicted by (*t* = 2.858; *p* = 0.004). It means that there is a positive linkage that exists between GI and GCI.

### 4.4. Mediation Analysis (Indirect Model)

In the context of this study, the mediating role of green image (GI) in the relationship between two predictors (SGMO and GSCM) and an outcome variable (GCI) has been investigated. For this purpose, a bootstrapping technique has been adopted in order to ascertain the indirect effects of GI in the relationship between SGMO, GSCM, and GCI. According to Santos (1999), the bootstrapping technique is the most effective and preferred procedure for the purpose of undertaking a mediation analysis [146].

The results of the mediation analysis have been presented in Table 6. It can be observed that the sixth Hypothesis H6 stands accepted as indicated by (*t* = 2.342; *p* = 0.020). This reveals that GI partially mediates the relationship between SGMO and GCI. In addition to this, the seventh Hypothesis H7 has also been accepted as suggested by (*t* = 2.717; *p* = 0.007). Therefore, it can be ascertained that GI mediates the relationship between GSCM and GCI. It signals that environmentally friendly products are more attractive to consumers when they allow the consumers to signal desirable personal status and display status, so signaling helps to overcome the gap between consumer and products [138]. Signalers can gain advantages in social interaction and consumers pay a premium price for environmentally friendly products [147].

### 4.5. Moderation Analysis

In the context of this study, the brand social responsibility (BSR) was taken as a moderator. Table 7 below depicts the moderating effect of BSR in the relationship between GI and GCI.

## 5. Discussion

This study was intended to examine and analyze the impact of strategic green marketing orientation and green supply chain management on green consumption intention with the mediating role of green image and the moderating role of brand social responsibility. A total of eight hypotheses were proposed and data was obtained from 317 consumers of the hotel and hospitality industry. The data were analyzed, applying partial least square structured equation modeling (PLS-SEM) using the SmartPLS software in order to ascertain the validity of the proposed hypotheses. The current study analyzed the proposed hypotheses through the lens of the signaling theory.

This section encompasses a detailed discussion of the results along with theoretical and managerial implications and directions for further research.

The first Hypothesis H1 envisaged that there was a positive relationship between strategic green marketing orientation (SGMO) and green consumption intention. The results indicated that a positive relationship did exist between the two constructs. Hence, it could be ascertained that an increase in the strategic green marketing orientation activities of the organization will lead to an increase in the development of green consumption intentions amongst the consumers. This finding supports the prior studies conducted in similar contexts. This finding is in complete harmony with the findings of who also examined the impact of SGMO on the purchase intentions of consumers [16]. The results suggested that the SGMO activities of the organizations drive the consumers to develop positive perceptions towards the firm, which in turn lead them to engage in the development of green consumption intentions. Similarly, Maheshwari (2014) also analyzed the impact of green marketing orientation on the purchase behavior of Indian consumers [97]. The results depicted that an increase in SGMO led to a significant and noticeable increase in green consumption intentions of the consumers.

The second Hypothesis H2 proposed a positive relationship between strategic green marketing orientation (SGMO) and a hotel’s green image (GI). The results of the study indicated the presence of a significant positive relationship between these two constructs. Therefore, it can be ascertained that an increase in the SGMO activities of the hotel will lead to a significant enhancement in the development of a hotel’s green image. This finding reinforces the findings of prior studies. For instance, Zhang et al. (2019), in their study on Chinese firms, analyzed the impact of green marketing activities on green brand image [120]. The results suggested that the green marketing initiatives taken by the firms led to the development of a green corporate image. Moreover, Bu et al. (2020) also analyzed the impact of environmental orientation on a firm’s performance and concluded by saying that the green environmental initiatives undertaken by a firm positively influence its overall performance and solidify its brand presence in the market [81]. According to signal theory, the hotel is the sender of signals and the consumers are the receivers of the signals [11].Therefore, it can be concluded that the green marketing orientation activities of the firm play a critical role in the development of a green corporate image.

The third Hypothesis H3 stated that there was a positive relationship between green supply chain management (GSCM) and green consumption intention (GCI). This hypothesis was accepted as the results suggested the presence of a positive association between GSCM and GCI. This means that an increase in green supply chain management initiatives will lead to a significant increase in the development of the green consumption intentions of the consumers. These findings are homogeneous with the findings of previous research. For instance, Ahmed et al. (2018) undertook a study on Bangladeshi manufacturing firms to investigate the impact of GSCM activities on the green consumption intentions of consumers. The results of the study revealed that green supply chain management activities of the organization led to the development of positive customer perceptions and these resulted in the development of purchase intentions. Similarly, Garcia et al. (2020), in their study also observed a positive linkage between GSCM and customer purchase intentions. Therefore, it can be concluded that green supply chain management is a major antecedent and precursor of green consumption intention.

The fourth Hypothesis H4 proposed that there was a positive relationship between green supply chain management (GSCM) and green image (GI). This hypothesis was also accepted as the results revealed that these two constructs were positively linked with each other. It means that an increase in the green supply chain management activities of a firm leads to the development of a positive green corporate image. This finding is completely homogeneous with the findings of prior studies. For instance, Aslam (2019) undertook a study on Pakistan firms to examine the impact of green supply chain management activities of the firms on the corporate image. The results of the study indicated that GSCM initiatives positively influenced the development of a strong and positive corporate image. These findings were also supported by [81]. Hence, it can be concluded that green supply chain management is an important factor that is a key predictor of green corporate image.

The fifth hypothesis H5 suggested a positive relationship between green image (GI) and green consumption intention (GCI). This hypothesis was also accepted as the results indicated the presence of a strong positive association between GI and GCI. These findings are consistent with the findings of previous studies carried out in similar contexts. For instance, Sarkis and Zhu (2018) carried out a study to determine whether green brand image influences the customers to develop green purchase intentions [13]. The results revealed that a green brand image led to the generation of positive customer perceptions, which in turn drove them to engage in purchase behavior. Similarly, Han and Huo (2020) in their study on Chinese apparel consumers revealed that green image (GI) played a major role in inducing the consumers to develop purchase intentions towards a particular brand [84]. Hence, it can be ascertained that green image is also a major precursor of green consumption intention.

The sixth and seventh hypotheses H6 and H7 proposed that a hotel’s green image (GI) mediates the relationship between strategic green marketing orientation (SGMO), green supply chain management (GSCM) and green consumption intention (GCI). The results confirmed the mediating role played by the green image in the relationship between SGMO, GSCM and GCI. The results were consistent with the previous studies that also confirmed the mediating role of green image. For instance, the impact of sustainable business practices on customer purchase intentions through the mediating role of green corporate image [16]. The results of the study confirmed that a green image mediated the relationship between sustainable business practices and customer purchase intentions. Therefore, it can be concluded that a green image is an important construct that can aid in explaining the relationship between green organizational initiatives and consumer behavioral outcomes.

Hypothesis eight H8 proposed that brand social responsibility (BSR) moderates the relationship between green image (GI) and green consumption intention (GCI). The results rejected this hypothesis and it was observed that BSR did not moderate the relationship between green image and green consumption intention. These results were in contrast with the findings of Fatma and Rahman (2016) who indicated that social responsibility plays the role of a catalyst in fostering green behavioral outcomes [139]. The reasons behind the rejection of this hypothesis can be associated with the fact that the Pakistani hotels displayed a complacent attitude towards the undertaking of their social responsibilities. Moreover, consumers tend to view a hotel’s social responsibility as just another publicity stunt or a marketing gimmick undertaken by firms to promote themselves. This leads to the development of a negative perception and as a result, the consumers tend to disengage themselves from depicting certain behaviors towards the firm’s offerings [7]. A firm can use brand social responsibility as a market signal to build some brand reputation [16,57,145,146].

The findings of the current study have enhanced the existing body of knowledge on green consumption intention in certain key dimensions. Firstly, the current study has played a crucial role in solidifying the current literature on the relationship between strategic green marketing orientation, green supply chain management and green consumption intention from the perspective of a visitor/tourist consumer. The current study also makes a crucial attempt to make a key contribution to an evolving area of research examining the aforementioned relationships in the Pakistani societal context. This study is one of the few that attempts to understand the antecedents of green consumption from the perspective of Pakistani tourist or hotel visitors in the northern regions of Pakistan.

Moreover, the findings of the current study suggest that a hotel’s green image is a major construct that aids in explaining the relationship between SGMO, GSCM and green consumption intention. Moreover, the green image has also been observed to be a major antecedent of green consumption intention. Hence, it can be ascertained that a hotel’s green image drives the consumers to engage in certain pro-environmental behaviors and outcomes such as green consumption intentions.

Lastly, the present study reinforces the current literature by analyzing the moderating role of a hotel’s social responsibility in the relationship between green image and green consumption intention. The results reveal that a hotel’s social responsibility does not moderate the development of green consumption intentions. This finding supports prior studies that suggested that social responsibility, if not done in the right manner, can prove to be counterproductive and can yield certain negative consumer behavioral outcomes such as disengagement, lack of trust and dissatisfaction. The research implications of the current study are categorized as a theoretical and practical perspective as described below.

### 5.1. Managerial Implications

The current study also extends some key implications for managers, marketing professionals and policy makers. The results of the study indicate the presence of a positive linkage between strategic green marketing orientation, green supply chain management and green consumption intentions. In order to promote the development of green consumption intentions, it becomes necessary for the managers and policy drafters to devise certain mechanisms and policies that are aimed at adopting green practices and approaches in order to mitigate the hotel’s overall carbon footprint. This can be done in certain ways, such as digitalization of the hotel’s processes, adopting green packaging, green transportation methods. Moreover, hotels should establish green practices in order to create a perception of a green brand in the eyes of their consumers. The development of positive consumer perceptions will greatly enhance the development of green consumption behaviors.

The findings of this study hold immense importance for organizations and marketing managers. It becomes imperative for the marketing managers to to develop a strategic green marketing strategy that is focused on projecting a green image for the hotel. The way in which this can be done is by highlighting the green initiatives undertaken by the hotels through various digital and electronic media. By doing this, hotels can convey their green initiatives to the consumers in an effective manner. This will generate a positive green image of the hotels which will, in turn, induce the consumers to develop consumption intention towards the hotel’s rooms, foods, and services.

### 5.2. Limitations of the Study and Future Research Directions

The present study encompasses a set of limitations. Firstly, the scope of the present study was limited to the visitors/tourists of the hospitality industry visiting the northern regions of Pakistan. Therefore, the results of the study may not be generalized to other industrial sectors. Hence, it becomes necessary to expand the scope of this study by increasing the sample size in order to improve generalizability. The time horizon of the present study was cross-sectional. Future studies could adopt longitudinal research designs in order to yield more reliable results. Moreover, this study only examined the mediating and moderating roles of green image and brand social responsibility. Future studies could introduce other mediating and moderating mechanisms in order to get a deeper insight into the factors that influence green consumption intention e.g., environmental concern, environmental responsibility, and environmental knowledge. These variables might include gender, income levels, and stakeholder pressures. Moreover, tactical and external green marketing orientation can be used as independent constructs in order to analyze their influence on green consumption intention. This will significantly aid in understanding the various antecedents and predictors of green consumption intention.

## 6. Conclusions

The current study examined and tested the theoretical framework shown in Figure 1 and the results completely supported the fact that strategic green marketing orientation and green supply chain have a significant positive effect on green customer purchase intention. A green image partially mediates the association between the said constructs, while corporate social responsibility does not moderate the linkage of green brand image and customer purchase intention.

The environment is facing serious challenges due to the increase in the processes of globalization and consumerism. Cut-throat competition, industrialization and an increase in products and services has had an adverse impact on the environment. This increased degradation has resulted in an alteration of consumer behavioral patterns. Consumers are now more informed than ever and they tend to develop perceptions and make judgments on the basis of the information conveyed to them through various signals received from organizations. Recent studies point towards the fact that consumers show an increased willingness towards the offerings of those hotels that adhere to the fulfillment of their environmental obligations. In contrast, consumers tend to disassociate themselves from those organizations that do not undertake any sort of green marketing initiatives. Hence, it becomes a matter of great importance for hotels to address the parameters that are focused on developing green marketing and supply chain strategies in order to engage in green consumption intention.

## Figures and Tables

**Table 1:** Demographic characteristics.

Demographic Characteristics	Frequency	(%)	Valid	Cumulative (%)
Gender				
Male	196	61.8	61.8	61.8
Female	121	38.2	38.2	100.0
Age				
20–30 years	139	43.8	43.8	43.8
31–40 years	150	47.3	47.3	91.2
41–50 years	20	6.3	6.3	97.5
51–60 above	08	2.5	2.5	100.0
Education				
High School/College	129	40.7	40.7	40.7
Undergraduate	118	37.2	37.2	77.9
Postgraduate	70	22.1	22.1	100.0
Income				
Below PKR 50,000	168	53.0	53.0	53.0
PKR 50,000 to 100,000	94	29.7	29.7	82.6
PKR 100,000 to 200,000	34	10.7	10.7	93.4
Above PKR 200,000	21	6.6	6.6	100.0
Total	317	100.0	100.0	

**Table 2:** Descriptive statistics.

Constructs	N	Mean	Std. Deviation	Skewness		Kurtosis	
	Statistic	Statistic	Statistic	Statistic	Std. Error	Statistic	Std. Error
SGMO	317	3.1951	0.67223	−0.463	0.137	−0.402	0.273
GSCM	317	3.4830	0.42069	−0.240	0.137	−0.435	0.273
GI	317	3.5902	0.84107	0.232	0.137	−0.174	0.273
BSR	317	3.2438	0.67895	−0.403	0.137	−0.284	0.273
GCI	317	3.2248	0.69707	−0.447	0.137	−0.871	0.273
Valid N (listwise)	317						

**Table 3:** Factor loadings, CA, CR and AVE.

ITEMS	FL	CA	CR	AVE
**SGMO**		0.793	0.795	0.562
SGMO1	0.651			
SGMO2	0.726			
SGMO3	0.587			
SGMO4	0.614			
SGMO5	0.792			
SGMO6	0.773			
**GSCM**		0.721	0.726	0.659
GSCM1	0.615			
GSCM2	0.721			
GSCM3	0.768			
GSCM4	0.643			
GSCM5	0.804			
GSCM6	0.691			
GSCM7	0.561			
GSCM8	0.595			
**GI**		0.868	0.905	0.712
GI1	0.836			
GI2	0.866			
GI3	0.868			
GI4	0.844			
GI5	0.618			
**BSR**		0.820	0.868	0.614
BSR1	0.918			
BSR2	0.797			
BSR3	0.743			
BSR4	0.724			
BSR5	0.753			
BSR6	0.668			
BSR7	0.691			
**GCI**		0.713	0.821	0.746
GCI1	0.787			
GCI2	0.826			
GCI3	0.796			
GCI4	0.690			

SGMO = strategic green marketing orientation; GSCM = green supply chain management; GI = green image; BSR = brand social responsibility; GCI = green consumption intention; Cronbach’s Alpha (CA); composite reliability (CR); average variance extract (AVE).

**Table 4:** Discriminant validity.

Variables	BSR	GCI	GI	GSCM	SGMO
BSR	**0.706**				
GCI	0.396	**0.737**			
GI	0.579	0.46	**0.812**		
GSCM	0.465	0.288	0.314	**0.807**	
SGMO	0.456	0.305	0.551	0.258	**0.635**

SGMO = strategic green marketing orientation; GSCM = green supply chain management; GI = green image; BSR = brand social responsibility; GCI = green consumption intention.

**Table 5:** Direct relationships.

Hypothesis/Path	Original Sample (O)	Standard Deviation (STDEV)	*t*-Statistics (O/STDEV)	*p*-Values	Decision
H1 SGMO -> GCI	0.199	0.073	2.739	0.006	Accepted
H2 SGMO -> GI	0.315	0.115	2.742	0.006	Accepted
H3 GSCM -> GCI	0.410	0.052	7.961	0.000	Accepted
H4 GSCM -> GI	0.502	0.072	6.967	0.000	Accepted
H5 GI -> GCI	0.173	0.061	2.858	0.004	Accepted

SGMO = strategic green marketing orientation; GSCM = green supply chain management; GI = green image; BSR = brand social responsibility; GCI = green consumption intention.

**Table 6:** Mediation analysis.

Hypothesis/Path	Original Sample (O)	Standard Deviation (STDEV)	*t*-Statistics (O/STDEV)	*p*-Values	Decision
H6 SGMO -> GI -> GCI	0.054	0.023	2.342	0.020	Accepted
H7 GSCM -> GI -> GCI	0.032	0.018	2.717	0.007	Accepted

**Table 7:** Moderation analysis.

Hypothesis/Path	Original Sample (O)	Standard Deviation (STDEV)	*t*-Statistics (O/STDEV)	*p*-Values	Decision
H8 BSR * GI -> GCI	0.061	0.039	1.566	0.118	Rejected

The eighth Hypothesis H8 has been rejected as indicated by (*t* = 1.566; *p* = 0.118). Hence, it can be ascertained that BSR does not moderate between GI and GCI.

## Data Availability

Not applicable.
